# *Cryptococcus neoformans*: Tripping on Acid in the Phagolysosome

**DOI:** 10.3389/fmicb.2016.00164

**Published:** 2016-02-17

**Authors:** Carlos M. DeLeon-Rodriguez, Arturo Casadevall

**Affiliations:** ^1^Department of Microbiology and Immunology, Albert Einstein College of Medicine, BronxNY, USA; ^2^Department of Molecular Microbiology and Immunology, Johns Hopkins University School of Public Health, BaltimoreMD, USA

**Keywords:** *Cryptococcus neoformans*, macrophage, phagolysosomal membrane damage, pH, Interferon γ

## Abstract

*Cryptococcus neoformans* (Cn) is a basidiomycetous pathogenic yeast that is a frequent cause of meningoencephalitis in immunocompromised individuals. Cn is a facultative intracellular pathogen in mammals, insects and amoeba. Cn infection occurs after inhalation of spores or desiccated cells from the environment. After inhalation Cn localizes to the lungs where it can be phagocytosed by alveolar macrophages. Cn is surrounded by a polysaccharide capsule that helps the fungus survive in vivo by interfering with phagocytosis, quenching free radical bursts and shedding polysaccharides that negatively modulates the immune system. After phagocytosis, Cn resides within the phagosome that matures to become a phagolysosome, a process that results in the acidification of the phagolysosomal lumen. Cn replicates at a higher rate inside macrophages than in the extracellular environment, possibly as a result that the phagosomal pH is near that optimal for growth. Cn increases the phagolysosomal pH and modulates the dynamics of Rab GTPases interaction with the phagolysosome. Chemical manipulation of the phagolysosomal pH with drugs can result in direct and indirect killing of Cn and reduced non-lytic exocytosis. Phagolysosomal membrane damage after Cn infection occurs both *in vivo* and *in vitro*, and is required for Cn growth and survival. Macrophage treatment with IFN-γ reduces the phagolysosomal damage and increases intracellular killing of Cn. Studies on mice and humans show that treatment with IFN-γ can improve host control of the disease. However, the mechanism by which Cn mediates phagolysosomal membrane damage remains unknown but likely candidates are phospholipases and mechanical damage from an enlarging capsule. Here we review Cn intracellular interaction with a particular emphasis on phagosomal interactions and develop the notion that the extent of damage of the phagosomal membrane is a key determinant of the outcome of the Cn-macrophage interaction.

## Cn as a Facultative Intracellular Pathogen

*Cryptococcus neoformans* (Cn), a basidiomycetous pathogenic yeast, is a relatively frequent cause of meningoencephalitis in immunocompromised individuals (Horgan et al., 1990; Thinyane et al., 2015). Cn is ubiquitous in the environment, inhabiting soils (Currie et al., 1994; Gugnani et al., 2005; Randhawa et al., 2008) and human infection occurs when aerosolized spores or desiccated fungal cells enter the lung via inhalation where Cn encounters the first line of defense: the alveolar macrophage (Feldmesser et al., 2000). Macrophages play a critical role in the pathogenesis of cryptococcosis, ranging from control of infection to possible roles in persistence, latency and extrapulmonary dissemination. Although historically Cn was divided into two varieties known as *neoformans* and *gattii*, genetic studies have subsequently separated these varieties into two species. The species *Cryptococcus gattii* has the potential to cause disease in immunocompetent individuals and animals (Stephen et al., 2002; Hoang et al., 2004). However, this review will only focus on Cn, since most of the macrophage interaction studies have been done with Cn. Cn is an facultative intracellular pathogen in such diverse hosts as mammals, amoebae (Steenbergen et al., 2001) and insects (Tenor et al., 2015; Trevijano-Contador et al., 2015), and employs various virulence factors to subvert cellular defense mechanisms. The manner in which Cn interacts with amoeba and macrophages is similar, suggesting that selection pressures in soil could lead to the emergence of particular traits that confer the capacity for virulence, thus making this microbe an accidental pathogen for mammals (Casadevall, 2012). In mammals, Cn was established to be a facultative intracellular pathogen *in vivo* and *in vitro* almost two decades ago (Feldmesser et al., 2001). In subsequent years, several groups have made major contributions to our understanding of the pathogenic strategy of Cn and those advances will be reviewed here.

The most distinctive feature of Cn is the expression of a large polysaccharide capsule that is a major virulence factor. The capsule functions in virulence through numerous mechanisms including preventing phagocytosis, quenching free radical bursts and interfering with immune responses (Bulmer and Sans, 1967; Zaragoza et al., 2008). Another mechanism by which Cn avoids phagocytosis is by the formation of titan cells, which prevent ingestion as a result of their enormous size (Okagaki et al., 2010; Zaragoza et al., 2010; Okagaki and Nielsen, 2012). The antiphagocytic function of the capsule is particularly relevant for intracellular pathogenesis since this process requires ingestion of the fungus by phagocytic cells. In the absence of opsonins, the capsule interferes with phagocytosis such that ingestion of encapsulated cells by macrophages is markedly lower (Macura et al., 2007). However, in the presence of capsule specific antibody and complement opsonins mediate efficient phagocytosis as described (Voelz and May, 2010). Although all encapsulated strains are opsonized by capsule binding antibodies, not all strains are efficiently opsonized by complement (Zaragoza et al., 2003). The mechanism for strain differences in complement opsonization involves differences in the geography of complement deposition in the capsule. If complement is deposited near the capsule surface, it is an effective opsonin, while complement deposition in the deeper layers of the capsule places complement component 3 in a location where it cannot interact with the complement receptor resulting in poor phagocytosis (Zaragoza et al., 2003). In addition, the capsule complement deposition pattern can be affected by the use of serum from different species, capsule size, and composition and the chronological age of the fungus (Young and Kozel, 1993; Gates and Kozel, 2006; Cordero et al., 2011). Therefore, the ability of Cn to increase its capsule size during infection is a mechanism that helps avoid complement-mediated phagocytosis.

**FIGURE 1:**
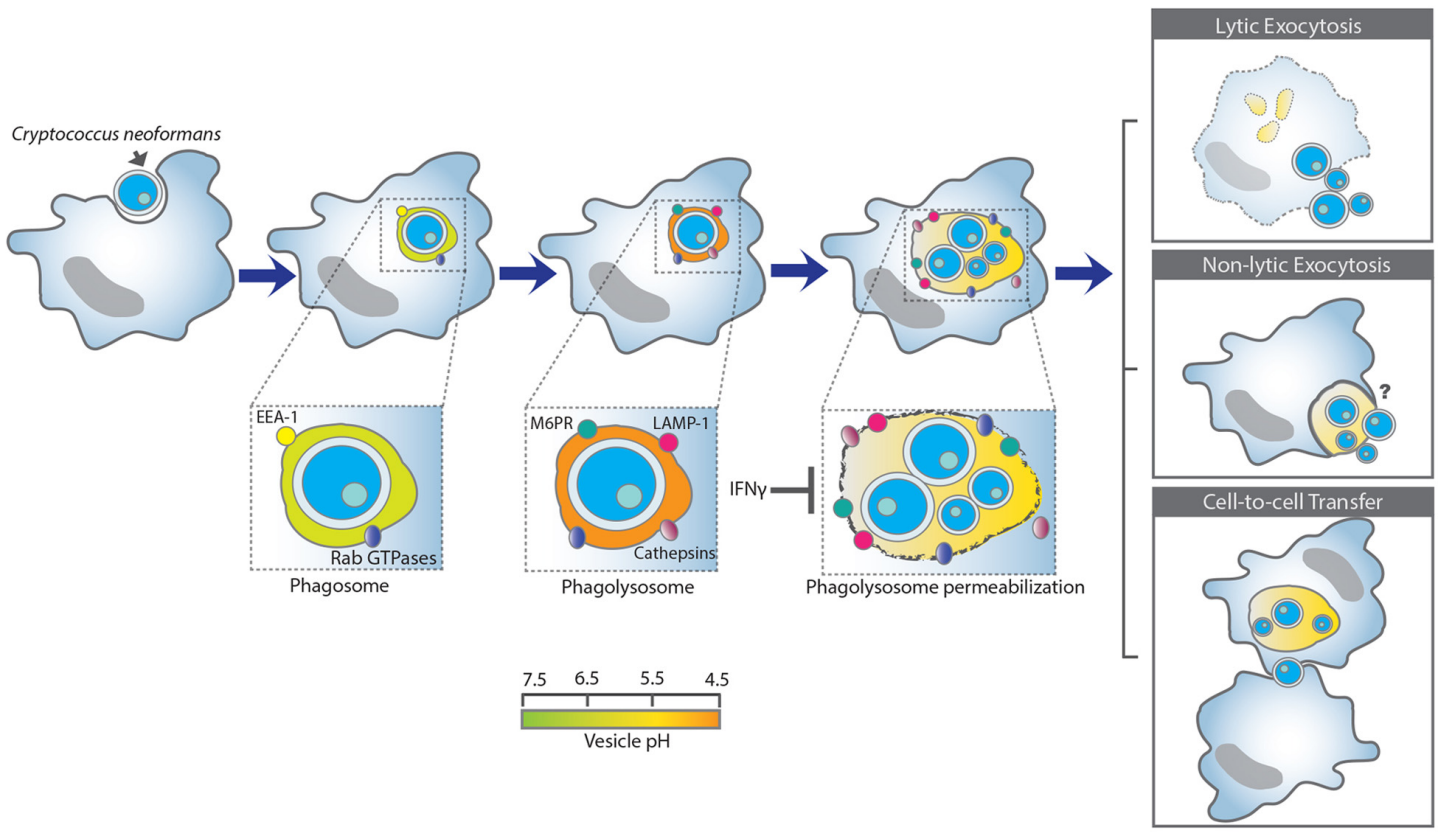
**A schematic summary of the *Cryptococcus neoformans* and macrophage interaction.** Upon internalization Cn reside within phagosomes that mature into phagolysosomes by interacting and fusing with the early- and late- endosome, and lastly with the lysosome. Cn-containing phagolysosomes acidify shortly to around pH 4.3 but increased later to 5.3, which is less acidic than the normal phagolysosomal of pH 4.5 (Levitz et al., 1999). Phagolysosomal membrane permeabilization could contribute to the increase in pH, possibly by affecting the proton gradient require to maintain acidification and/or promoting leakage of contents into the cytoplasm and vice-versa. Phagosomal membrane damage favors Cn replication and survival. In contrast, IFN-γ reduces phagolysosomal membrane damage and promotes Cn killing (Davis et al., 2015). Phagosomal membrane integrity appears to be a key determinant of whether Cn lives or dies after ingestion. Intracellular Cn survival and replication can have three major outcomes: (1) lysis of the macrophage and release of Cn; (2) non-lytic exocytosis in which both the macrophage and Cn survive (non-lytic exocytosis can be complete or partial), and (3) cell-to-cell transfer in which one macrophage can pass a previously ingested Cn to another macrophage.

Cn is able to survive and replicates at a higher rate inside macrophage than in the extracellular environment (Diamond and Bennett, 1973; Feldmesser et al., 2000). This ability of Cn to survive and replicate inside macrophages correlates with the virulence of clinical isolates, and is associated with dissemination via a Trojan horse hypothesis whereby Cn can cross the blood brain barrier inside macrophages (Charlier et al., 2009; Alanio et al., 2011). After infection, Cn can persist in the host in a latent state inside macrophages and multinucleated giant cells in granulomas. Cn in this latent state can emerge and cause disease if the host immune status change from immunocompetent to immunocompromised (Shibuya et al., 2005; Saha et al., 2007; Alanio et al., 2015). The macrophage-Cn interaction can have three major outcomes: (1) intracellular killing of Cn or control growth by the macrophage; (2) lysis of the macrophage and release of Cn; and (3) non-lytic exocytosis in which both the macrophage and Cn survive (**Figure 1**). In addition, the phenomenon of macrophage to macrophage transfer of Cn cells has been described *in vitro* (Alvarez and Casadevall, 2007; Ma et al., 2007; Stukes et al., 2014). Of these possibilities exocytosis is the most common outcome (Stukes et al., 2014). Depletion of alveolar macrophage in rats and mice shows that the role of macrophages during Cn infection varies with the host species. Rat macrophages controlled Cn intracellular growth and were more resistant to pathogen-mediated lysis. When rat lung macrophages were depleted the animals became more vulnerable (Shao et al., 2005). In contrast, murine macrophage served as a replicative niche for Cn and growth of Cn inside the macrophage can result in lysis of the macrophage (Shao et al., 2005). Differences in mouse strain susceptibility to Cn infection correlates with macrophage permissiveness for fungal intracellular replication (Zaragoza et al., 2007) but these differences are not well understood.

Acidification of the phagosome is an important macrophage antimicrobial mechanism but certain pathogens are able to adapt to overcome or exploit these acidic environments (Criscitiello et al., 2013). Pinpointing the mechanism by which Cn is able to survive within the harsh environment of the phagolysosome can provide insight to develop ways to reduce the intracellular replication of Cn and potentially prevent/treat disease. During infection, Cn experiences different pH levels and ability to survive in the host requires survival in the slightly alkaline environment of the blood and the cerebrospinal fluid, and the acidic environment of the phagolysosome. Consequently we will review the studies that explore the interactions of Cn in these diverse ranges of pH and phagosomal maturation.

## Cn Localization into the Phagolysosome

The localization of the microbe in tissue can play an important role on the virulence and survival of the pathogen. In tissues, such as lungs, that mount granulomatous responses, Cn is often found inside macrophages or in close approximation to them (Yamaoka et al., 1996; Kobayashi et al., 2001). To survive in their hosts, pathogenic microbes implement different strategies to avoid killing and degradation by the phagocytic cell, including inhibiting phagosome maturation (Clemens et al., 2000) or phagolysosome fusion (Horwitz, 1983), blocking phagosomal acidification (Horwitz and Maxfield, 1984) or escaping from the phagosome (Gaillard et al., 1987). By examining the localization of known endosomal and lysosomal markers, any inhibition of phagosome maturation or phagolysosome fusion during Cn infection can be observed. Studies done with Cn-infected human monocyte-derived macrophage and J774.16 macrophage-like cells showed co-localization of the yeast with the lysosomal-associated membrane protein (LAMP-1), a lysosomal marker (Levitz et al., 1999; Alvarez and Casadevall, 2006). Cn-containing phagosomes in murine bone marrow derived dendritic cells and monocyte derived human dendritic cells fused with the early endosome marker (EEA-1) and LAMP-1 as early as 10 minutes after incubation with Cn (Wozniak and Levitz, 2008). *In vivo* studies also showed that phagolysosomal fusion occurred by two hours after infection in the alveolar macrophage in a murine model (Feldmesser et al., 2000). Furthermore, studies with bone marrow derived dendritic cells expressing CD63-mRFP1 and Class II MHC-eGFP determined that these markers are recruited to Cn-containing vacuoles. Thus, class II MHC proteins are recruited before CD63 and CD63 recruitment is dependent on the acidification of the phagosome (Artavanis-Tsakonas et al., 2006). In contrast, LAMP-1 recruitment is independent of phagosome acidification (Artavanis-Tsakonas et al., 2006). Cryptococcal phagolysosomes have some characteristics of autophagomes such as manifesting LC3 (microtubule-associated protein 1 light chain 3 alpha) co-localization (Nicola et al., 2012).

Cn intracellular trafficking is conserved in *Drosophila melanogaster* S2 cells and J774.A1 cells, upon internalization by both cell types Cn co-localized with EEA-1, the late endosome marker, mannose-6-phosphatase receptor (M6PR), LAMP-1 and Cathepsin D, another lysosomal marker (Qin et al., 2011). Furthermore, surfactant protein D was shown to enhance phagocytosis of acapsular cryptococcal mutants by macrophages *in vitro* but the resulting Cn-containing phagolysosome shows a decrease in co-localization with LAMP-1 (Geunes-Boyer et al., 2009), suggesting that surfactant protein D influences Cn-containing phagosome maturation. Based on these studies, it was generally thought that Cn resided in the phagolysosome upon internalization by the macrophage and did not interfere with phagosome maturation. However, new evidence based on the analysis of the Rab GTPases localization, which are early endosomes and phagosomes markers, revealed a different pattern for the interaction of these molecules with phagosomes containing live Cn, heat killed Cn or latex beads, suggesting that Cn influenced phagosome maturation. Rab 5 and 11 are recruited to the Cn-containing vesicle shortly after phagocytosis but their presence diminishes more rapidly in phagosomes containing live fungal cells than in those containing latex beads- or heat killed Cn-cells (Smith et al., 2015). However, the extent to which Cn affects phagosome maturation is unknown but it is likely that the effect is smaller than other pathogens, like *Mycobacteria tuberculosis*, which directly interfere with phagosome maturation by blocking fusion with late endosomes and lysosomes (Kelley and Schorey, 2003).

Phagosomal maturation and acidification are required for both the localization and optimal enzymatic activity of lysosomal proteases, respectively (Boya and Kroemer, 2008). Cn localization in the phagolysosome exposes the yeast to cathepsins, other hydrolases, and reactive oxygen species in the lysosomal lumens. Cathepsins are lysosome related proteases with a role in lysosomal protein recycling, Toll-like receptors signaling, extracellular matrix degradation, activation and inhibition of cytokines, antigen processing, and more recently have been implied in apoptosis. To activate apoptosis, cathepsins need to be released from the lysosomes into the cytosol via lysosomal membrane permeabilization (Conus and Simon, 2010). Lysosomal extracts purified from bone marrow derived dendritic cells can kill Cn in a dose dependent manner (Wozniak and Levitz, 2008). Analysis of purified lysosomal enzymes showed that Cathepsin B and Cathepsin L inhibited Cn growth, and that an inhibitor of Cathepsin B enzymatic activity surprisingly increased inhibition of Cn growth (Hole et al., 2012). These results suggested that Cn exposure to lysosomal proteases, specifically cathepsin B and L inhibited cryptococcal growth. *In vivo* analysis of Cathepsins B and Cathepsin L activity in Cn-containing phagolysosome has shown little evidence of enzymatic activity, but macrophages challenged with heat killed Cn show Cathepsin L activity (Smith et al., 2015). The discrepancy between these results might be attributed to the fact that in the first studies Cn was incubated *in vitro* with lysosomal extracts whereas in the later studies the analysis was done in infected cells. The Cn capsule confers resistance against reactive oxygen species (Zaragoza et al., 2008) and presumably protects the fungal cell during the lysosomal oxidative burst. However, as to whether the capsule also provides resistance against cathepsins and others proteases remain unclear. In this regard, the dense polysaccharide fibrillar network that surrounds the cell could provide protection by trapping cytotoxic enzymes but this mechanism remains to be shown.

In summary, the difference between cathepsin activity in phagosomes containing live and dead Cn suggests that Cn can affect cathepsin enzymatic activity but the mechanism is unknown. Given that phagosomes containing live Cn become progressively leaky during intracellular residence, one possible explanation for the discordance between the results obtained with live and dead Cn is that the negative cathepsin activity observed with the live cells is lost with a degraded phagosomal membrane.

## Effect of pH on the Cn Growth (Studies Independent of Host)

The response of Cn to pH is important in pathogenesis because the fungus goes from neutral to slightly alkaline conditions in extracellular body fluids to acidic conditions in the mature phagolysosomal compartment. An inverse correlation between growth of Cn and the pH of the growth medium was initially established more than 60 years ago (Mosberg and Mc, 1951; Howard, 1961), when several investigators reported that acidic milieus enhanced growth while alkaline conditions inhibited growth. More recent studies have confirmed those results by showing that Cn can grow in the pH range of 5–8 and also revealed that the optimal growth for Cn is at pH 5 (Levitz et al., 1997). These studies also established differences in the susceptibility of Cn strains to pH and a dependence on temperature such that fungal cells were less resistant at 37° to alkaline pH. However, a Cn strain deficient in the Ca^+2^-regulated protein Calcineurin is more susceptible to alkaline pH, and become avirulent in a rabbit model of cryptococcal meningitis (Odom et al., 1997). Interestingly, a Cn strain lacking glycosphingolipid glucosylceramide was avirulent in a murine animal model when challenged intranasally but was virulent when the infection was done intravenously, suggesting that the lung environment controlled the infection. The glycosphingolipid glucosylceramide mutant was arrested in the S and G_2_/M phase at pH 7.4 in 5% CO_2_, but is not affected at pH 4 in 5% CO_2_ (Rittershaus et al., 2006). One explanation for these observations is that during the intranasal infection the glycosphingolipid glucosylceramide mutant is not able to replicate in the alkaline extracellular environment of the lung. Those fungal cells that are ingested by the alveolar macrophage can replicate in the acidic environment of the phagolysosome but these are controlled by the formation of granulomas. In contrast, after intravenous infection the glycosphingolipid glucosylceramide mutant growth was also arrested in the alkaline environment of the blood, but was able to replicate once it invaded other organs and reside in abscess with an acidic environment optimal for the growth of the mutant (Rittershaus et al., 2006). These studies suggest that the ability of Cn to grow at different pH is important for pathogenicity.

The conserved Rim101 pathway mediates fungal response to extracellular neutral/alkaline pH, for reviews see (Davis, 2009; Cornet and Gaillardin, 2014). Extensive work has been done to identify the homologues of the Rim101 pathway and the role of Rim101 pathway during Cn–host interaction. Cn Rim101 mutants show a capsule defect, altered cell wall composition and increased susceptibility to different host induced stress stimulus (O’Meara et al., 2014). Recently, the Rim pathway was shown to be activated by the increase of pH, with the protein Rra1 functioning as a pH sensor, but others components of the pH sensing complex have not been identified (Ost et al., 2015). Further studies of this pathway may shed light unto how Cn is able to modulate gene expression to survive between the different pH environments it encounter during host infection.

## Cn and Phagolysosomal Acidification

Phagosome maturation results in the acidification of the phagosomal lumen, creating an optimal environment for the proteases recruited to that site during the maturation process. In the next two sections, we review what is known about phagosomal acidification during Cn infection of phagocytic cells and the effect of chemical modulation of phagosomal acidification on the outcome of Cn infection. Initial studies measuring *in vivo* phagolysosomal pH done in rabbit alveolar macrophages after 24 h infection using fluorescein-labeled heat-killed cryptococcal cells revealed an average pH 5 and 5.2 for phagolysosomes containing heat killed Cn and fluorescein-labeled silica particles, respectively. They noted that approximately 2% of the phagolysosomes containing heat-killed Cn had a pH of 6.5, which never occurred with the silica particles (Nessa et al., 1997). The pH of Cn-containing phagolysosomes was also measured in monocyte-derived macrophage using live Cn and heat-killed Cn after 3 and 24 h infection. The pH of phagolysosomes containing heat-killed Cn remained stable over time, ranging from 5.2 to 5.1, but the pH of phagolysosomes containing live Cn increased from 4.3 to 5.3, which closely matches the optimal pH for fungal growth. The phagolysosomal pH of neutrophils infected with live Cn remained constant over a 3 h period hovering between 5.2 and 5.0 (Levitz et al., 1999). A more recent study suggests that live Cn, but not heat-killed Cn, could block acidification of the phagolysosome (Smith et al., 2015). The differences in these studies could reflect the use of different cell models, Cn strains, and/or technical approaches. Cn mutants in phospholipase B, Sec14 secretion system, urease expression, and the acapsular mutant maintained their ability to prevent acidification, suggesting that prevention of acidification occurred through an independent process that is unrelated to those virulence factors (Smith et al., 2015). However, a strain of Cn that overexpressed the antifungal resistance protein 1 (AFR1) delayed the acidification of the phagolysosome and resided in phagolysosomes with a lower degree of co-localization with Rab5-, Rab7- and LAMP2 (Orsi et al., 2009). In summary, Cn growth is affected by pH but the acidity of the phagolysosomal compartment does not appear to be a significant mechanism for microbial control and may in fact promote fungal replication.

## Cn and Chemical Manipulation of Phagosomal Acidification

Experiments done in the late 1990s showed that treatment of BV2 microglial cells with the weak bases, chloroquine, and ammonium chloride, enhanced the anticryptococcal activity of microglial cells. These weak bases act as lysosomotropic agents by accumulating in the lysosomal compartment (Villamil Giraldo et al., 2014). Similar effects were observed when microglial cells were treated with bafilomycin A1, an inhibitor of the vacuolar-type H^+^-ATPases. These investigators also demonstrated an increase in the median survival time of mice treated with an intracerebral administration of chloroquine before challenge with a lethal dose of Cn (Mazzolla et al., 1997). Similar results were noted when treating human monocyte-derived macrophages (MDM) with chloroquine and ammonium chloride, which increased anticryptoccocal activity of the MDM independent of iron deprivation. Chloroquine enhancement of anticryptococcal activity was also observed with monocytes derived from HIV-seronegative and HIV-seropositive donors, and in a murine model of experimental cryptococcosis using immunocompetent and immunodeficient mice (Levitz et al., 1997). Chloroquine treatment increased the pH of phagosomes containing heat-killed Cn in a dose dependent manner showing pH values of approximately 5.2 at 1 μM, 6.5 at 10 μM, and 7.5 at 100 μM (Levitz et al., 1999). Interestingly, both chloroquine and quinacrine accumulate within Cn and directly inhibit its growth. Accumulation of chloroquine and quinacrine within Cn increased at physiological conditions, but the mechanism by which it exerts its anticryptococcal activity remains unknown (Harrison et al., 2000). Cn growth was also inhibited by ammonium chloride and bafilomycin A in a concentration dependent manner (Harrison et al., 2000). These results suggest that treatment of phagocytic cells with lysosomotropic agents had a direct effect on Cn as well as an alternative indirect effect by increasing the pH of the Cn-containing phagolysosome (Harrison et al., 2000; Weber et al., 2000).

Non-lytic exocytosis can occur after phagocytosis of Cn by macrophages, and results on the expulsion of viable Cn to the extracellular environment without the lysis of the macrophage. Blockage of phagosome maturation using Concanamycin A, an inhibitor of V-ATPase, reduced Cn non-lytic exocytosis (Ma et al., 2006). Macrophage treatment with bafilomycin A revealed a slight decrease in non-lytic exocytosis, while treatment with ammonium chloride and chloroquine significantly increase non-lytic exocytosis (Ma et al., 2006; Nicola et al., 2011; Qin et al., 2011). The mechanism for how pH affects non-lytic exocytosis remains unexplained.

## Cn and Phagolysosome Membrane Permeabilization or Damage

As a consequence of lysosomal membrane permeabilization, cathepsins and other proteases are released from the lysosomal lumen into the cytosol where they can activate cell death (Boya and Kroemer, 2008). Phagolysosomal membrane damage was observed in alveolar macrophage of mice infected with Cn at 7 days post-infection (Feldmesser et al., 2000). Subsequent studies revealed that Cn-containing phagosomes pre-loaded with fluorescently labeled dextran showed diffusion of the fluorescent signal, indicative of leakage of phagosomal contents into the cytoplasm. Lysosomal membrane permeabilization was confirmed by demonstrating the inability of the Cn-containing phagosome to maintain an acidic environment (Tucker and Casadevall, 2002). The mechanisms responsible for phagolysosomal permeability are unknown. Cn extracellular phospholipase activity was hypothesized to have a role on the degradation of the phagolysosomal membrane but this effect was not experimentally demonstrated (Cox et al., 2001). Nonetheless, Cn phagolysosomal damage is associated with cryptococcal replication and survival, but activation of macrophages with IFN-γ can reduce phagolysosomal damage (Davis et al., 2015). In Cn-infected THP-1 macrophage-like cells, phagolysosomal membrane permeabilization induces formation of the adaptor protein apoptosis-associated speck-like protein containing a CARD speck, suggesting that release of phagolysosomal content, including Cathepsin B, activates inflammasomes resulting in processing and release of IL-1β. Treatment with Cathepsin B inhibitor reduced IL-1β secretion implying that phagolysosomal damage is required for activation of the canonical caspase-8 inflammasome (Chen et al., 2015). Taken together, internalized Cn induces phagolysosomal membrane permeabilization and leads to host cell death in a manner dependent on inflammasomes activation.

## Conclusion and Perspective

During the preparation of this review, it was apparent that the literature is inconsistent with how it refers to the phagolysosome and the timing of its appearance. According to LAMP-1 staining, the Cn-containing phagosome fuses with the lysosome as early as one hour post incubation, implying that Cn resides inside the phagolysosome by one-hour post ingestion. To avoid confusion during this review, we used the term phagolysosome whenever we referred to studies that used experimental time of one-hour post incubation or longer. Readers should note that phagosomal maturation is a dynamic process that may vary between individual phagosomes. For example, EEA1 and LAMP-1 were each associated with some phagosomes at early time of macrophage infection but the number of phagosomes positive for these markers increased gradually with time (Wozniak and Levitz, 2008). Phagosomal acidification is a critical step during phagosomal maturation to allow phagosome-lysosome fusion and provide an optimal environment for the activity of antimicrobial enzymes. However, internalization of Cn results in a decrease of phagolysosomal pH shortly after ingestion, which is followed by an increase in phagolysosomal pH over time culminating in an inability of the Cn-containing phagolysosome to maintain the acidic pH as a result of membrane damage (Levitz et al., 1999; Tucker and Casadevall, 2002). Further studies are needed to determine the precise relationship between changes in pH in the Cn-containing phagolysosome, Cn growth and the onset of phagolysosomal membrane damage. Future studies should take in consideration the effect of phagolysosomal membrane permeabilization with regards to the acidification of the Cn-containing phagolysosome and determined causal and temporal relationships, if any. If phagolysosomal membrane permeabilization occurs only in a fraction of those Cn-containing phagolysosome, it is possible that will result in a gradient of pH values, as damage of the phagolysosomal membrane will result in neutralization of phagolysosome acidification by cytoplasmic contents.

Phagolysosome membrane damage can promote Cn growth after cell ingestion (Davis et al., 2015), presumably by disabling microbicide mechanisms or damaging the host cells through the spillage of vesicular content into the cytoplasm. The mechanism for Cn induction of phagolysosome membrane damage remains unknown but various hypotheses have been discussed in the literature. These include the notion that phagolysosomal membrane damage is a result of Cn replication and capsular growth that produce physical damage (Feldmesser et al., 2000). Secondly, secreted fungal proteins damage the phagolysosomal membrane directly. In this regard, extracellular phospholipase was suggested as a candidate for phagolysosomal membrane damage (Cox et al., 2001). Phospholipids induce enlargement of Cn capsule which also requires phospholipase B activity (Chrisman et al., 2011). It is possible that there is a synergistic effect that combines damage of the phagolysosomal membrane by the extracellular fungal phospholipase activity and physical damage of the phagolysosomal membrane by growth of the fungal capsule induced by the phospholipase products.

Cn intracellular residence was shown to result in damage to a variety of cellular systems including mitochondrial function (Coelho et al., 2015). The amount of damage incurred by the host cell may depend on the degree of cellular activation. In this regard, treatment of macrophage with IFN-γ was shown to protect the phagolysosomal membrane from damage and promoted the anti-fungicidal ability of the macrophages (Davis et al., 2015). Previous studies shows that IFN-γ also increased anti-fungicidal activity in rat alveolar macrophage and natural killer cells, and increase survival time in two murine models of Cn infection (Mody et al., 1991; Salkowski and Balish, 1991; Kawakami et al., 1995, 1996). IFN-γ administrations were shown to prolong mice survival when used as an adjuvant treatment in combination with amphotericin B in normal mice and SCID mice infected with Cn (Joly et al., 1994; Lutz et al., 2000; Clemons et al., 2001). IFN-γ has been used clinically as adjuvant treatment and was shown to improve in Cn clearance from the cerebrospinal fluid in HIV-positive patients with cryptococcal meningitis (Jarvis et al., 2012). These observations in rodents and humans suggest that interventions that promote phagosomal membrane integrity could have potential therapeutic applications.

In summary, it appears that the phagolysosomal membrane is a key battleground in the struggle between Cn and phagocytic cells. Damage to the membrane with loss of acidity and spillage of phagolysosomal contents into the cytoplasm favors the fungus and catalyzes as series of events that compromise the host cells and interfere with their ability to control infection. On the other hand, integrity of the phagolysosomal membrane is associated with control of infection. At this time the factors that tip the balance toward membrane damage or integrity are poorly understood and their elucidation is a research priority in the field. The understanding of this process is given additional urgency since therapeutic interventions to stabilize the phagolysosome may tip the balance to the benefit of the host.

## Author Contributions

CD-R and AC collaborated to write the manuscript.

## Conflict of Interest Statement

The authors declare that the research was conducted in the absence of any commercial or financial relationships that could be construed as a potential conflict of interest.
